# Multi-Omics Analysis Reveals the Systematic Relationship Between Oral Homeostasis and Chronic Sleep Deprivation in Rats

**DOI:** 10.3389/fimmu.2022.847132

**Published:** 2022-03-31

**Authors:** Pan Chen, Hao Wu, Hongliang Yao, Jiashuo Zhang, Weiyang Fan, Zhen Chen, Weiwei Su, Yonggang Wang, Peibo Li

**Affiliations:** ^1^Guangdong Engineering and Technology Research Center for Quality and Efficacy Re-evaluation of Post-marketed Traditional Chinese Medicine (TCM), State Key Laboratory of Biocontrol, Guangdong Provincial Key Laboratory of Plant Resources, School of Life Sciences, Sun Yat-sen University, Guangzhou, China; ^2^Guangdong Key Laboratory of Animal Conservation and Resource Utilization, Guangdong Public Laboratory of Wild Animal Conservation and Utilization, Institute of Zoology, Guangdong Academy of Sciences, Guangzhou, China

**Keywords:** chronic sleep deprivation, oral inflammation, oral microbiota, oral homeostasis, multi-omics analysis

## Abstract

Sleep disorders were associated with oral health. Inflammation has especially been thought to be a key factor in linking oral diseases and sleep deficiency. However, how chronic sleep deprivation (CSD) affects oral homeostasis, particularly oral inflammation and oral microbiota, is still unknown. This study aimed to uncover the systematic relationship between oral homeostasis and CSD in rats. The metabolomics in serum, proteomics in the tongue tissues, and microbiome analysis in the oral cavity in CSD rats were performed. Multi-omics data integration analysis was performed to uncover the systematic relationship between oral homeostasis and CSD through the weighted correlation network analysis. We found that CSD could lead to oral inflammation in rats. CSD significantly increased systemic inflammation by enhancing the serum levels of IL-1β, IL-6 and inhibiting the serum level of IL-10. Serum levels of adrenocorticotropin hormone, corticosterone, and triiodothyronine were increased in CSD rats, and the steroid hormone biosynthesis pathway was also found to be involved in the perturbation resulting from CSD, together suggesting the activation of the hypothalamic-pituitary-adrenocortical and hypothalamic‐pituitary‐thyroid axis. CSD led to changes of oral microbiota composition, and g_*Acinetobacter*, *Candidatus Chryseobacterium massiliae*, and g_*Moraxella* were significantly correlated with multiple proteins in bacterial invasion of epithelial cells pathway, which may partially responsible for oral inflammation resulting from CSD. The changes of proteomic profiling expression caused by CSD in tongue tissues were mainly enriched in neurodegenerative diseases pathways and immune/inflammation-related pathways. Multi-omics analysis indicated that the inflammatory response-related modules were significantly correlated with the neurodegenerative disease-related module suggesting a possible link between neurodegenerative diseases and oral inflammation. Together, CSD induced oral inflammation and subtle changes on oral microbiota. Our study is helpful to further understand the role that oral homeostasis plays in the process by which CSD affects human health and disease.

## Introduction

Sleep is a physiological behavior that is closely related to human health. However, chronic sleep deprivation (CSD) is becoming more prevalent due to health conditions, sleep disorders, work requirements, and lifestyle changes. Sleep deficiency produces physiological consequences that are harmful to endocrine functions, metabolic functions, and inflammatory responses (1). Lack of sleep has also been found to raise the risk of neurodegenerative diseases (2), cardiovascular diseases (3), hypertension (4), diabetes (5), obesity (6), colitis (7), and pregnancy complications (8).

Sleep disorders were reportedly associated with oral health (9, 10). Carra et al. reported that the individuals with self-report sleep disorders have increased risk of gingival inflammation based on a cross-sectional study (9). Sleep disorders can be influenced by craniofacial morphology and can in turn affect oral health (10). To date, little is known about how sleep disorders affect oral health conditions. The potential risk caused by sleep disorders for oral health seem to be directly related to the inflammatory and immune alterations resulting from sleep disorders (i.e., sleep deprivation or poor sleep quality) (9). Our previous study found that sleep deficiency could exacerbate mouth ulcers and delay their healing process (11). Several studies supported the associations between poor oral health and chronic systemic diseases such as diabetes mellitus, neurodegenerative diseases, and atherosclerosis (12, 13). It can thus be seen that oral health and sleep deficiency are both strongly associated with systemic chronic diseases, such as neurodegenerative diseases. It is reasonable to point out that studying the systemic effects of sleep deprivation on oral homeostasis can help further understand how sleep deficiency affects human health and disease. However, how CSD affects oral homeostasis, especially oral inflammation and oral microbiota, is still unknown. It would therefore be of high significance to investigate the effects of CSD on oral homeostasis.

Inflammation has particularly been considered as a critical factor that connects oral diseases and chronic systemic diseases (14). Oral inflammation is a common physiological immune response that if persists may produce damage as well as increase the risk of progression to lesions (15). In addition, the oral mucosa may be accompanied by a low-grade chronic upregulation of inflammatory mediators that may be associated with microbes (16). A previous study proposed that certain low abundance microbial pathogens can interfere with the host immune system and remodel the microbiota, leading to oral inflammatory diseases such as gingivitis and periodontitis (17). It has been reported that the oral cavity is an active microbial environment during sleep and that changes in oral environmental conditions could have a large impact on the absolute microbial abundances observed (18). However, no studies on the association between sleep deprivation and oral microbes could be found. In this study, we aimed to uncover the systematic relationships between oral homeostasis and CSD in rats.

## Materials and Methods

### Animals

Male Sprague-Dawley rats (~220 g, 8-week-old) from the Laboratory Animal Center of Sun Yat-sen University were housed in a standard laboratory environment with a 12/12-h light-dark cycle and were supplied with food and water *ad libitum*. Before the experiments began, the animals were habituated to maintenance conditions for one week to avoid environmental stress. The protocols of animal experiments were approved by the IACUC of Sun Yat-sen University (Approval No. SYSU-IACUC-2020-000383) and performed in strict compliance with the institutional guidelines.

### Procedure for Producing CSD and Study Design

CSD was described as previous studies using a water tank device with multiple platforms (19, 20). The rats were housed in an individual tank (110 cm × 70 cm × 30 cm) with 15 platforms (6.3 cm in diameter) rising 1 cm above the water surface, and can move freely. During each episode of rapid eye movement (REM) sleep, animals were awakened due to loss of postural muscle tone falling into the water, which abolished REM sleep and also led to the loss of approximately 30% of non-REM sleep (21). All animals were acclimatized to a sleep deprivation environment for 0.5 h per day for one week prior to the onset of sleep deprivation. Water bottles and chow pellets are placed on a grid at the top of the tank to allow the animals to drink and eat freely. The animals were randomly assigned into the control group and the CSD group (n = 12 per group). The rats in the CSD group were placed in the water tank at 16:00 h until 10:00 h, then placed back in their home cages, resulting in 18 h/day of sleep interruption. The water in the tanks was changed with clean water daily. To control the differences in environmental conditions, the rats in the control group were adapted to the CSD environment for 0.5 h/day at16:00 h and housed in the home-cage where they slept freely for the remaining time. This cyclic procedure was continued for 21 days. The diagram of the study design is shown in **Supplementary Figure S1**.

### Sample Collection

At the end of the CSD period, a sterile swab (greiner bio-one, German) was swiped five times along with the tongue of each rat from back to front. Swab samples were stored at –80°C. Then, blood is collected from the abdominal aorta after all the animals were anesthetized. The blood was centrifuged (5,000 rpm, 4°C, 20 min) to obtain serum, which was then stored at −80°C. The whole-brain, tongue, and buccal mucosa tissues were collected and frozen promptly at −80°C

### Enzyme-Linked Immunosorbent Assay

The serum concentrations of interleukin (IL)-1β, IL-6, IL-10, adrenocorticotropin hormone (ACTH), corticosterone (CORT), and triiodothyronine (T3) were determined using ELISA kits according to the operating instructions. All commercial ELISA kits were purchased from the Nanjing Jiancheng Bioengineering Institute (Jiangsu, China).

### Measurement of Neurotransmitter Levels

The levels of 5-hydroxytryptamine (5-HT), g-aminobutyric acid (GABA), and norepinephrine (NE) in serum and whole-brain tissues were measured using ultra-high performance liquid chromatography-tandem mass spectrometry as described in our previous study (22).

### Histological and Immunohistochemistry Analyses

The mucosal samples fixed in 4% paraformaldehyde (BioSharp, Anhui, China) were embedded in paraffin. Sections were cut in 4 μm in thickness for hematoxylin and eosin (H&E; Aladdin, Shanghai, China) staining. The sections were analyzed using an optical microscope (Olympus BX43; Olympus Co, Tokyo, Japan). Immunohistochemistry was performed to determine the expression of IL-6 and IL-1β in buccal mucosa tissues as described in our previous study (22). The anti-IL-6 primary antibody (1:500, NB600-1131, Novus, CO, USA) and IL-1β primary antibody (1:500, NB600-633, Novus, CO, USA) were used. Images of these sections were obtained using a Nikon ECLIPSE E100 optical microscope (Nikon Co., Tokyo, Japan). The integrated optical density (IOD)/area was used to represent the average optical density of each sample section using Image-Pro Plus 6.0.

### Oral Microbiome Analysis

The advanced third-generation sequencing (TGS) technology was applied for oral microbiome analysis. Compared with the sequencing technology of 16s rRNA, TGS technology has an advantage of longer reads, while allowing it to detect isolated genomic DNA without amplification and produce high quality genomic assemblies (23). Oral bacterial genomic DNA from the frozen swab samples was extracted using a PowerSoil DNA Isolation kit. The full-length 16S rRNA gene was PCR-amplified using the bacterial primers 27F (5′-AGRGTTYGATYMTGGCTCAG-3′) and 1492R (5′-RGYTACCTTGTTACGACTT-3′). PCR amplification was performed using a reaction volume consisting of 10 μL buffer, 4 μL dNTP, 1 μL of each primer (10 μM), 0.4 μL DNA polymerase, and 50 ng genomic DNA. The PCR was set up as previously described (24). The electrophoresed PCR products were purified, quantified, and homogenized to create a SMRTbell sequence library.

Circular consensus sequencing (CCS) reads were obtained using the SMRT Link v8.0 with minPasses ≥ 5 and minPredictedAccuracy ≥ 0.9. CCS reads were barcode-identified and length-filtered using Lima v1.7.0; the chimeras were removed thereafter using UCHIME 8.1. The Optimized-CCS reads were clustered by USEARCH 10.0 to generate operational classification units (OTUs) based on 97% sequence similarity. The sequences were annotated using the SILVA bacterial 16S rRNA database. The detected communities were identified and annotated at different taxonomic levels.

The alpha diversity of oral microbiota was analyzed using Chao1, Simpson, Shannon, and ACE methods. The principal co-ordinates analysis (PCoA) was applied to evaluate beta diversity of oral microbiota based on the Binary-Jaccard distances; the permutational analysis of variance (PERMANOVA) analysis was utilized. Linear discriminant analysis (LDA) effective size (LEfSe) analysis was applied to search for biomarkers that were statistically different between the control and CSD groups using Galaxy online tool (http://huttenhower.sph.harvard.edu/galaxy/). At the species level, the microbes with the LDA score > 2 and P < 0.05 (Wilcoxon rank-sum test) were considered to be differentially abundant taxa. Spearman correlation analysis between differential abundance bacteria and changed indexes was performed using R studio.

### Metabolomics Analysis of Serum

The untargeted metabolomics analysis of serum was performed with minor alterations as described in our previous study (22). Each serum sample (100 µL) was added to 200 µL of cold acetonitrile/methanol solution (1∶1, v/v) to precipitate the proteins. The mixtures were vortexed for 5 min and then centrifuged at 13,000 rpm (4°C, 20 min). Then, 10 µL of the supernatant was injected into the LC/MS (Shimadzu CBM20Alite controller/AB Sciex Triple TOF™ 5600+) system for analysis. The mass spectrometry data were acquired in negative ion mode and positive mode, respectively, and the parameter settings were described in our previous study (22).

One-MAP/PTO software (Dalian Dashuo Information Technology Co. Ltd., China) was used to detect and extract the peak data. Then, the metabolites were annotated by an in-house MS2 database and Human Metabolome Database (HMDB, https://hmdb.ca/). The metabolomics data were analyzed by orthogonal partial least squares discriminant analysis (OPLS-DA) using SIMCA (version 15.0.2, Umea, Sweden). Furthermore, the value of variable importance (VIP) was calculated in the OPLS-DA model, which indicates the importance of each metabolite. Metabolites were considered to be differential metabolites when VIP >1 and P < 0.05 (Student’s t-test). In addition, the KEGG (http://www.genome.jp/kegg/) and MetaboAnalyst (http://www.metaboanalyst.ca/) databases were used for pathway enrichment analysis.

### Proteomics Analysis of Tongue Tissue

Six tongue tissue samples from each group were randomly selected for proteomic analysis. The tongue samples were homogenized in 0.5 mL RIPA buffer for 4 min and then were staved with ultrasonic for 2 min in an ice bath. After centrifugation of the lysate (12000 rpm, 10 min, 4°C), the supernatant was collected. The total protein of the supernatant was quantified BCA method. The distilled water was added into 100 μg protein extracts of each sample with a final concentration of 1 mg/mL. The pre-chill acetone (-20°C, 5 times the volume of the sample) was added to the sample, mixed and then precipitated overnight at -20°C. After centrifugation of the mixture (12000 rpm, 10 min, 4°C), the supernatant was removed. Each sample was co-incubated with 5 mL of dithiothreitol (200 mM) for 1 h at 55 C, followed by 15 min for the room temperature co-incubation of iodoacetamide (10 mM). The digestion of the sample was conducted overnight with trypsin (Promega, Madison, WI, USA) in a 50∶1 mass ratio. The digested peptide product was labeled using TMT kits. Subsequently, peptides were desalted by C18 disks (Sigma­Aldrich, St. Louis, MO, USA) and vacuum-dried and separated using Waters XBridge BEH C18 XP Column (150 mm × 2.1 mm) to obtain high­PH fractionation peptides. A reversed­phase column (Reprosil-Pur 120 C18-AQ) was used for the separation of each fraction sample in a nano­UPLC system (EASYnLC1200). The mass spectral analysis was performed using a Q Exactive HFX Orbitrap instrument (Thermo Fisher Scientific) with a nano­electrospray ion source. The gradient elution program (A: 80% acetonitrile containing 0.1% formic acid; B: water containing 0.1% formic acid) was as follows: 0–2 min, 2 to 5% A; 2–70 min, 5% to 22% A; 70–86 min, 22% to 45% A; 86–88 min, 95% A.

For MS1, data dependent acquisition was performed in positive mode with a resolution of 120k and m/z range of 350­1600; For MS2, the fixed first mass was set to 110 m/z and the resolution was set to 45k. Proteome Discoverer software (V2.4.0.305) and a built-in Sequest HT search engine were applied to process the Vendor’s raw MS files. The proteomics data were analyzed by the OPLS-DA model using SIMCA (version 15.0.2, Umea, Sweden). Proteins with fold change > 1.2 or fold change < 0.83, and P < 0.05 (Student’s t-test) were considered as differentially expressed proteins. Metascape online tool (https://metascape.org/) was used to performed Gene Ontology (GO) and KEGG pathway enrichment analyses. The protein-protein interaction (PPI) analysis was conducted through STRING database (https://string-db.org/).

### Multi-omics Integration Analysis

For multi-omics data integration analysis, we implemented the weighted correlation network analysis (WGCNA) using the “WGCNA 1.70-3” R package (25). The soft-thresholding powers of 8 and 13 were chosen using the scale-free topology criterion for metabolomics and proteomics data respectively and used to calculate the unsigned correlation network adjacency. Network construction and module identification were implemented in the blockwiseModules function. The minimum module sizes for the identification of metabolite modules and protein modules were set as 5 and 30 respectively. An expression profile for each module was summarized as an eigenvalue. Thus, the module eigenvalue represented the overall variation of the module expression levels and was used for further analyses. We studied the relationships and similarities among the found modules by correlating the eigenvalues. We correlated eigenvalues with external traits (biochemical indices) and look for the most significant associations. Differentially expressed proteins that were clustered into a WGCNA module were subjected to KEGG pathway enrichment analysis using the Metascape database (https://metascape.org/). The network was constructed *via* the exportNetworkToCytoscape function in R and exported to Cytoscape_3.7.2. The hub proteins and hub metabolites in each module network were explored using the cytoHubba plugin (26).

### Statistical Analysis

Data analyses were performed using R studio and GraphPad Prism 8.0.2 (La Jolla, CA, USA). The results are presented as means ± SD for each group. Unpaired *t* test was carried out to assess the differences between the groups. Results were considered significant at P < 0.05. Details on statistical tests, n numbers, and significance cutoffs can be found in the figure legends. When relevant, further details are found in the method details for the specific measurement.

## Results

### Histopathology

**Figure 1:**
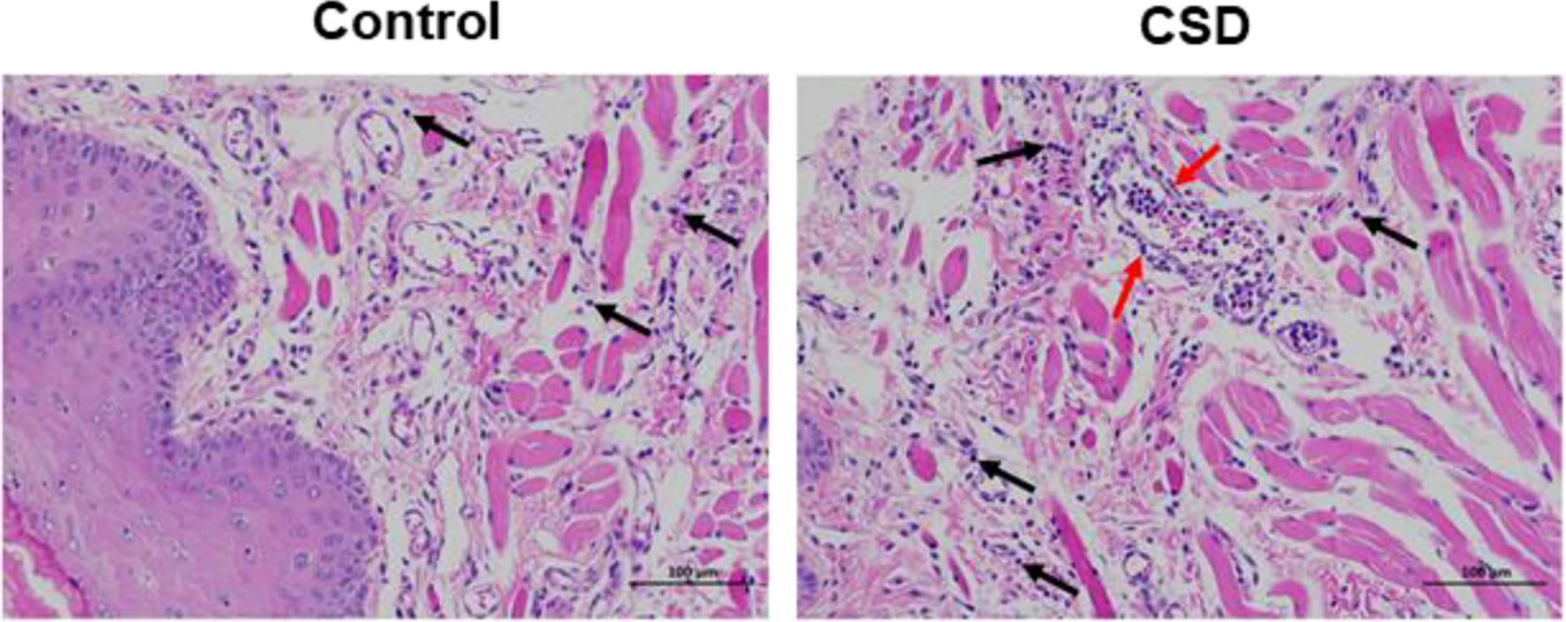
The effects of CSD on buccal mucosa in rats (Mucosal histopathology changes in rats). The results showed a more serious inflammatory histological appearance compared to the control group. The pictures were taken at 200×. The black arrows indicate infiltration of inflammatory cells and the red arrows indicate a large number of leukocytes in the lumen of the vessel, and a ring of inflammatory cells infiltrating around the vessel.

Histopathology results are shown in **Figure 1**. The histology of the buccal mucosa in both the control and CSD groups was fully covered by well-keratinized stratified epithelium. However, the homogeneous connective tissue in the CSD group was infiltrated with more inflammatory cells compared with the control group.

### Effects of CSD on IL-6 and IL-1β Expression in Buccal Mucosa Tissues

**Figure 2:**
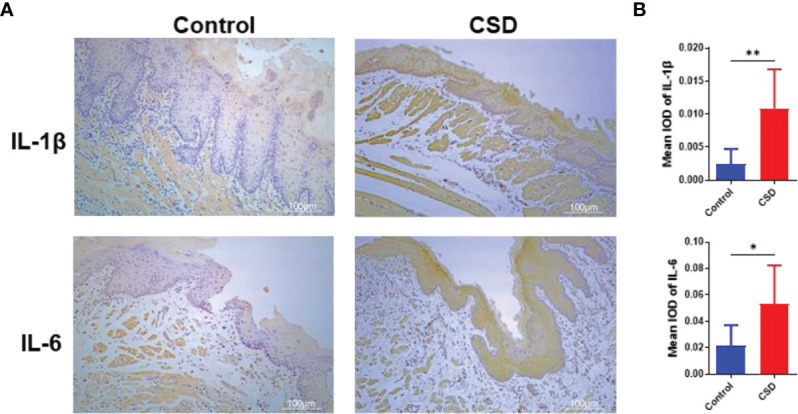
**(A)** Interleukin (IL)-1β and IL-6 expressions in epithelial tissue sections of buccal mucosa by immunohistochemistry in the control group and CSD groups. **(B)** Quantification of IL-1β and IL-6 expression in epithelial tissue section of the buccal mucosa. Statistical significance was analyzed by Student’s *t* test (n = 6, *P < 0.05; **P < 0.01).

To investigate the effects of CSD on oral inflammation, the expression of IL-6 and IL-1β in epithelial tissue sections was investigated by immunohistochemistry analysis. As illustrated in **Figure 2**, the expressions of IL-6 and IL-1β in epithelial tissue sections from the CSD group were significantly increased when compared with that of the control group.

### Effects of CSD on Cytokine, Neurotransmitter, and Endocrine Levels

**Figure 3:**
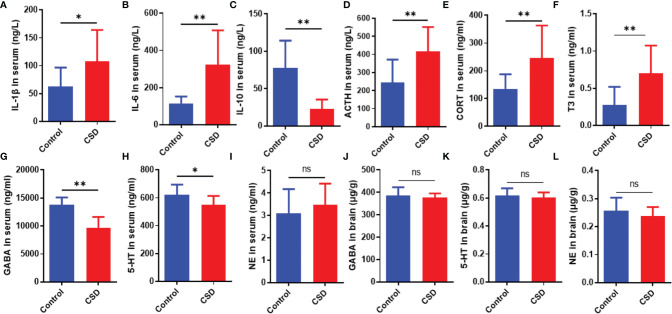
The effects of CSD on the cytokine, neurotransmitter, and endocrine levels. Serum levels of **(A)** IL‐1β, **(B)** IL‐6, **(C)** IL‐10, **(D)** ACTH, **(E)** CORT, **(F)** T3, **(G)** GABA, **(H)** 5-HT, and **(I)** NE, and brain levels of **(J)** GABA, **(K)** 5-HT, and **(L)** NE (n = 12). Data are expressed as means ± SD. Statistical significance was analyzed by Student’s *t* test (ns, no significance; *P < 0.05; **P < 0.01).

As shown in **Figures 3A–C**, CSD significantly increased the serum levels of IL-6, and IL-1β; while, CSD lowered the serum level of and IL-10. As shown in **Figures 3G–H**, CSD decreased the 5-HT and GABA levels in serum. However, compared with the control group, CSD did not lead to a significant change in the 5-HT and GABA levels in brain tissue, and the NE levels both in brain tissue and serum have no significant changes (**Figures 3J–L**). As shown in **Figures 3D–F**, CSD resulted in a significant increase in the serum level of ACTH, CORT, and T3. The above results demonstrated that CSD may active the hypothalamic-pituitary-adrenocortical (HPA) axis and hypothalamic‐pituitary‐thyroid (HPT) axis. However, the serum levels of IgM, IgG, and IgA did not show a significant change between that in the control group and the CSD group.

### The Effects of CSD on Oral Microbiota

**Figure 4:**
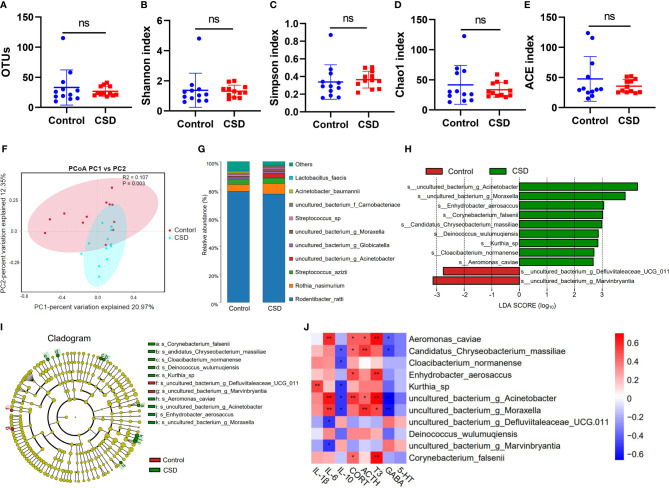
Impact of CSD intervention on oral microbiota. Responses of the alpha diversity of the oral microbiota to CSD in rats including **(A)** OTUs, **(B)** Shannon index, **(C)** Simpson index, **(D)** Chao1 index, and **(E)** ACE index of each group (n = 12, using unpaired Student’s *t* test; ns, no significance) **(F)** PCoA plots explaining 21% and 12% of the variance of OTUs adjusted for abundance of the control and CSD groups (PERMANOVA test). **(G)** Oral microbiota composition. **(H)** LDA scores of differentially abundant taxa between the control and CSD groups using the LEfSe method. **(I)** Taxonomic cladogram generated from the LEfSe analysis. **(J)** Spearman correlation analysis between differential abundance bacteria and changed biochemical indexes. (*P < 0.05; **P < 0.01).

In order to more closely examine the impact of CSD on the oral microbiome, we next examined the changes in the microbiota composition in response to CSD intervention. There are no significant changes at the OTU level between the control group and the CSD group (**Figure 4A**). The Chao1, Simpson, Shannon, and ACE indices did not reveal any significant differences (**Figures 4B–E**) between the control and CSD groups indicated that alpha diversity was not altered by CSD. PCoA results representing *β* diversity showed that oral microbiota overall composition in the CSD group was significantly different from that in the control group (PERMANOVA test, P = 0.003) (**Figure 4F**). Taxonomic classification at species level showed that the top three of the oral bacteria detected were *Rodentibacter*, *Rothia*, and *Streptococcus* (**Figure 4G**). The linear discriminant analysis (LDA) effect size (LEfSe) analysis was performed to identify the bacterial-specific taxa associated with CSD. The results of LEfSe analysis showed that abundance of oral microbiota in the CSD group exhibited 9 species increased and 2 species decreased compared with that in the Control group (**Figure 4H**). The taxonomic cladograms (**Figure 4I**), visualized the effects of CSD on the oral microbiota at the species level in rats. The results showed that the predominant bacteria in the oral of CSD rats were g_*Acinetobacter*, g_*Moraxella*, s_*Enhydrobacter aerosaccus*, s_c*orynebacterium falsenii*, s_*Candidatus Chryseobacterium massiliae*, s_*Deinococcus wulumuqiensis*, s_*Kurthia*_sp, s_*Cloacibacterium normanense*, and s_*Aeromonas caviae*. However, the abundances g_*Defluviitaleaceae*_UCG-011 and g_*Marvinbryantia* were decreased in CSD rats.

The results of Spearman correlation analysis between differential abundance bacteria and changed biochemical indexes are shown in **Figure 4J**. We found that *Kurthia* sp. was positively correlated with IL-1β; *Kurthia* sp. can infect tissue culture cells and has been found in the dental plaques of a beagle dog (27). *Aeromonas_caviae* was positively correlated with IL-6, T3, CORT, and ACTH, and was negatively correlated with GABA; it has been reported that *Aeromonas* in sleep deprivation‐induced intestinal barrier dysfunction in mice was increased and melatonin suppressed this increase (28). *Candidatus Chryseobacterium massiliae* was positively correlated with ACTH and CORT, and was negatively correlated with IL-10 and GABA. *Enhydrobacter aerosaccus* was positively correlated with CORT and T3; *Enhydrobacter aerosaccus* has been reported to be detected from a patient with haemophagocytic lymphohistiocytosis who was using corticosteroids (29). *Corynebacterium falsenii* was positively correlated with T3 and CORT, and was recognized as a primary pathogen (30). *Acinetobacter* was positively correlated with IL-6, CORT, T3, and ACTH, and was negatively correlated with GABA and IL-10. g_*Moraxella* was positively correlated with IL-6, ACTH, and T3, and was negatively correlated with GABA and IL-10. g_*Marvinbryantia* and g_*Defluviitaleaceae_UCG-011* was negatively correlated with IL-6; g_*Marvinbryantia* spp. could inhibit the host inflammation by producing SCFAs (31); It has been reported that the abundance of g_*Defluviitaleaceae_UCG-011* was decreased in oral of rheumatoid arthritis patients (32).

### Metabolomics Analysis of Serum

**Figure 5:**
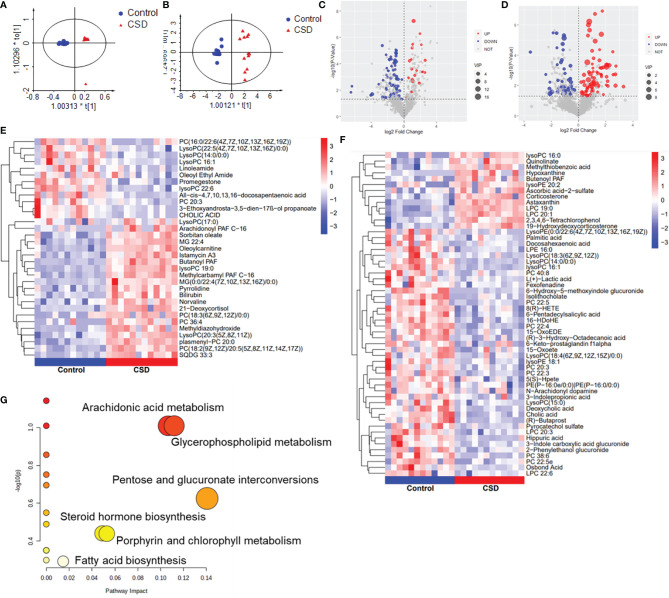
The effects of CSD on serum metabolic profiling in rats. Orthogonal partial least squares discriminant analysis (OPLS-DA) score plot for the control group vs the CSD group in **(A)** negative mode and in **(B)** positive mode. Volcano plots for the control group vs the CSD group in **(C)** positive mode and **(D)** negative mode. Heat map of the hierarchical clustering analysis of the differential metabolites for the control group vs the CSD group in **(E)** positive mode and **(F)** negative mode; the color represents the relative level of the differential metabolite with a gradient from red (high levels) to blue (low levels). **(G)** Metabolic pathway analysis results based on the differential metabolites.

**Figure 6:**
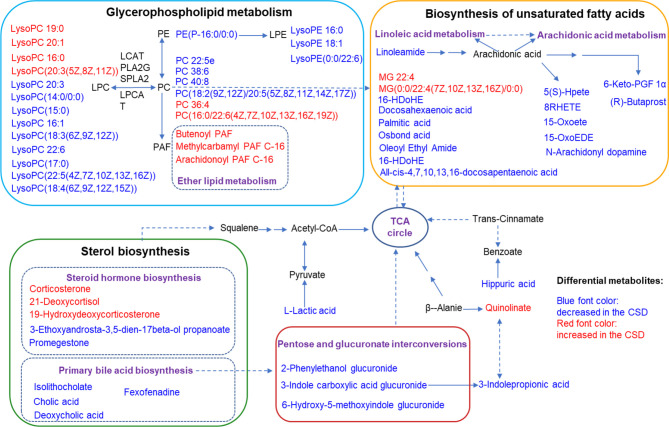
Summaries of metabolic pathways based on KEGG database. Blue font color represents the metabolites decreased in CSD rats, and red font color represents the metabolites increased in CSD rats.

Serum metabolic profiling in the control CSD groups was assessed by multivariate analysis. A clear separation was observed between the control and CSD groups in OPLS-DA score plots (**Figures 5A, B**), suggesting differential metabolic profiles between the two groups. The VIP and p-value of the metabolites between the control and CSD groups were visualized through volcano plots (**Figures 5C, D**). A total of 84 differential metabolites (**Supplementary Table 1**) were identified between the control and CSD groups, using a P-value < 0.05 (two-tailed Student’s *t*-test) and a VIP > 1.0 in the OPLS-DA models. The relative contents of the differential metabolites in each sample are presented as a heat map (**Figures 5E, F**). The metabolic pathway analysis using Metaboanalyst based on the KEGG database (Organism: Rat) revealed that six metabolic pathways were associated with CSD as shown in **Figure 5G**. The important differential metabolites were summarized in **Figure 6**.

### Proteomics Analysis of Tongue Tissue

**Figure 7:**
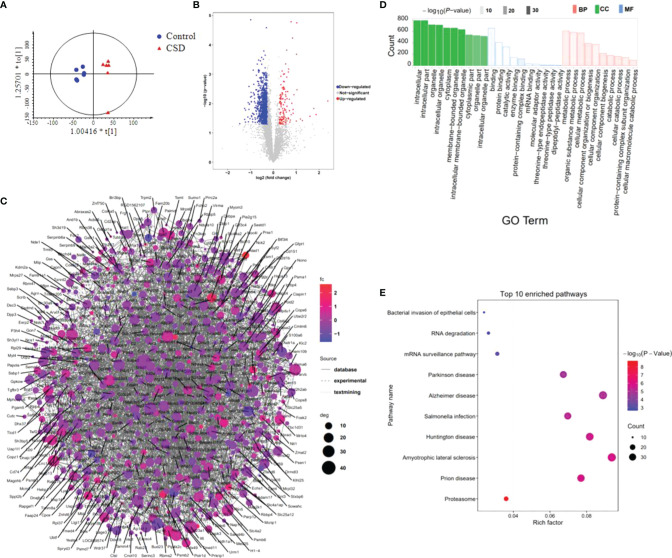
The effects of CSD on proteomic profiling of tongue tissue in rats. **(A)** OPLS-DA model shows a separation of clusters between the control group and CSD group. **(B)** Volcano plots for the control group vs the CSD group. **(C)** The differentially expressed proteins were searched in the STRING database to visualize potential protein networks. **(D)** Gene Ontology (GO) and **(E)** KEGG pathway enrichment analysis of the differentially expressed proteins.

TMT-based proteomics was performed to screen the differentially expressed proteins in tongue tissue between the control and CSD groups. OPLS-DA result exhibited an obvious separation of clusters between the control and CSD groups (**Figure 7A**). As shown in volcano plots (**Figure 7B**), a total of 977 differentially expressed proteins were identified (fold change > 1.3 or < 0.83, and p < 0.05, **Supplementary Table 2**). Among these 977 proteins, 263 proteins were upregulated and 714 were downregulated in the CSD group. The PPI network of differentially expressed proteins is shown in **Figure 7C** and **Supplementary Figure S2** (The PPI network data is shown in **Supplementary Table 3**). Mapk3, Rbm8a, Il6, Psmb6, and Nip7 were highly top-ranked by the degree in the PPI network.

The differentially expressed proteins were applied to perform GO and KEGG pathway enrichment analyses through the Metascape online tool. The results of GO analysis (**Supplementary Table 4**) show that 80 of 786 biological processes, 86 of 226 cell components, and 8 of 160 molecular functions enriched for these proteins were recognized as adjust-P < 0.05. The top 10 significantly enriched GO terms are shown in **Figure 7D**. According to the results of KEGG pathway enrichment, a total of 47 pathways have been identified in the KEGG database (Organism: Rat) with the P-value < 0.05 (**Supplementary Table 5**). The top 10 enriched pathways are shown in **Figure 7E**. The neurodegenerative diseases pathways including Amyotrophic lateral sclerosis, Prion disease, Huntington disease, Alzheimer disease, and Parkinson disease were enriched. Among these top 10 pathways, the bacterial invasion of epithelial cells pathway was related to oral ulcers. Ten proteins involved the bacterial invasion of epithelial cells pathway including Arpc1b, Sept8, Cav3, Pxn, Arpc5, Cd2ap, Crkl, Elmo1, Cbl, and Wasl. Another interesting focus is immune inflammatory responses occurred in the tongue tissues. We found that Proteasome, T cell receptor signaling pathway, B cell receptor signaling pathway, and TNF signaling pathway were significantly enriched by the differentially expressed proteins.

### Multi-Omics Integration Analysis

**Figure 8:**
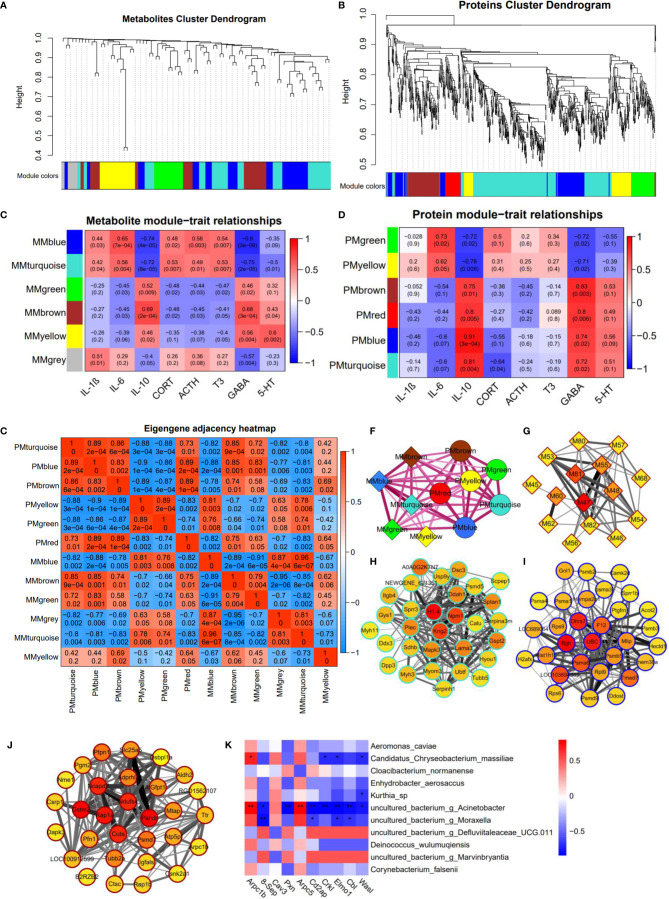
Multi-omics integration analysis through WGCNA. **(A)** Clustering dendrograms based on **(A)** metabolomic data and **(B)** proteomic data. Heatmap of the correlation between **(C)** metabolite and **(D)** protein module eigenvalues and biochemical traits. The correlations between protein modules and metabolite modules **(E)**, and are displayed as a network overlaid with adjacency values **(F)**. Network of **(G)** metabolites in MMbrown module, top 30 ranked proteins in **(H)** PMturquoise module, **(I)** PMblue module, and **(J)** PMbrown module; the color represents the MCC scores of the node with a gradient from yellow (low scores) to red (high scores). **(K)** The heat-map shows the Spearman correlation coefficients between proteins enriched in the bacterial invasion of epithelial cells pathway and oral bacteria (*P < 0.05; **P < 0.01).

We reasoned that integration of the clustered data could be used to identify relationships between proteins and metabolites. Therefore, we examined the integrated dataset for protein-metabolite relationships using the WGCNA R package. The clustering dendrograms based on metabolomic and proteomic data are shown in **Figures 8A, B**. For metabolite modules identification, a total of 5 metabolite modules (MMs) were generated, with four unassigned metabolites (grey module). Of these, the metabolite module green (MMgreen) is most highly negatively correlated with 8-OHdG; the MMblue is most highly positively correlated with IL-6 (**Figure 8C**). For protein modules identification, a total of 6 protein modules (PMs) were generated. Of these PMs, the PMblue module is most positively correlated with IL-10; PMblue is significantly correlated with CORT (**Figure 8D**). To identify the relationships between protein and metabolite modules, the correlation analysis was performed using the eigenvalues of each module. Many correlations between protein modules and metabolite modules were found (**Figure 8E**), which can be displayed as a network overlaid with adjacency values (**Figure 8F**). Among these correlations, PMturquoise, PMblue, and MMbrown modules are highly related. The co-regulation observed between these proteins and metabolites suggests that these biomarkers function in a similar pathway and highlight potential crosstalk between the multi-omics data. The result of the KEGG enrichment analysis of each PM network is shown in **Supplementary Table 6**. PMturquoise module is significantly linked to immunoinflammatory-related pathways. PMbrown module is significantly linked to neurodegenerative disease pathways.

8(R)-HETE is a top-ranked metabolite in the MMbrown network using the MCC algorithm through Cytoscape (**Figure 8G**). H1-4 is a top-ranked protein in the PMturquoise network (**Figure 8H**); Bgn is a top-ranked protein in the PMblue network (**Figure 8I**); Gstm2 and Parvb are the top-ranked proteins in the PMbrown network (**Figure 8J**). These top-ranked biomarkers selected from the network were also highly ranked in the binary comparison analysis. Most of these metabolites in MMbrown are involved in arachidonic acid metabolism (M47: 8(R)-HETE, M48: 15-OxoEDE, M45: 5(S)-Hpete, M46: 15-Oxoete, M54: 16-HDoHE). PMbrown module was significantly enriched in Alzheimer’s disease pathway, and highly related to PMturquoise module and MMbrown module which were both related to inflammation responses, suggesting that oral inflammation may play an important role in the emergence and progression of neurodegenerative diseases. Among the top-ranked proteins in the PMbrown network, Ndufs4 and Slc25a5 are involved in Alzheimer’s disease pathway, showing that they play an important role in neurodegenerative diseases resulting from sleep disorders. To explore the relationship between the microbiome and protein expression in oral cavity, we used a Spearman correlation analysis to examine the relationships between the proteins enriched in the Bacterial invasion of epithelial cells pathway and differential abundance bacteria. We found that g_*Acinetobacter*, g_*Moraxella*, and *Candidatus Chryseobacterium massiliae* were highly correlated with multiple proteins in bacterial invasion of epithelial cells pathway (**Figure 8K**).

## Discussion

**Figure 9:**
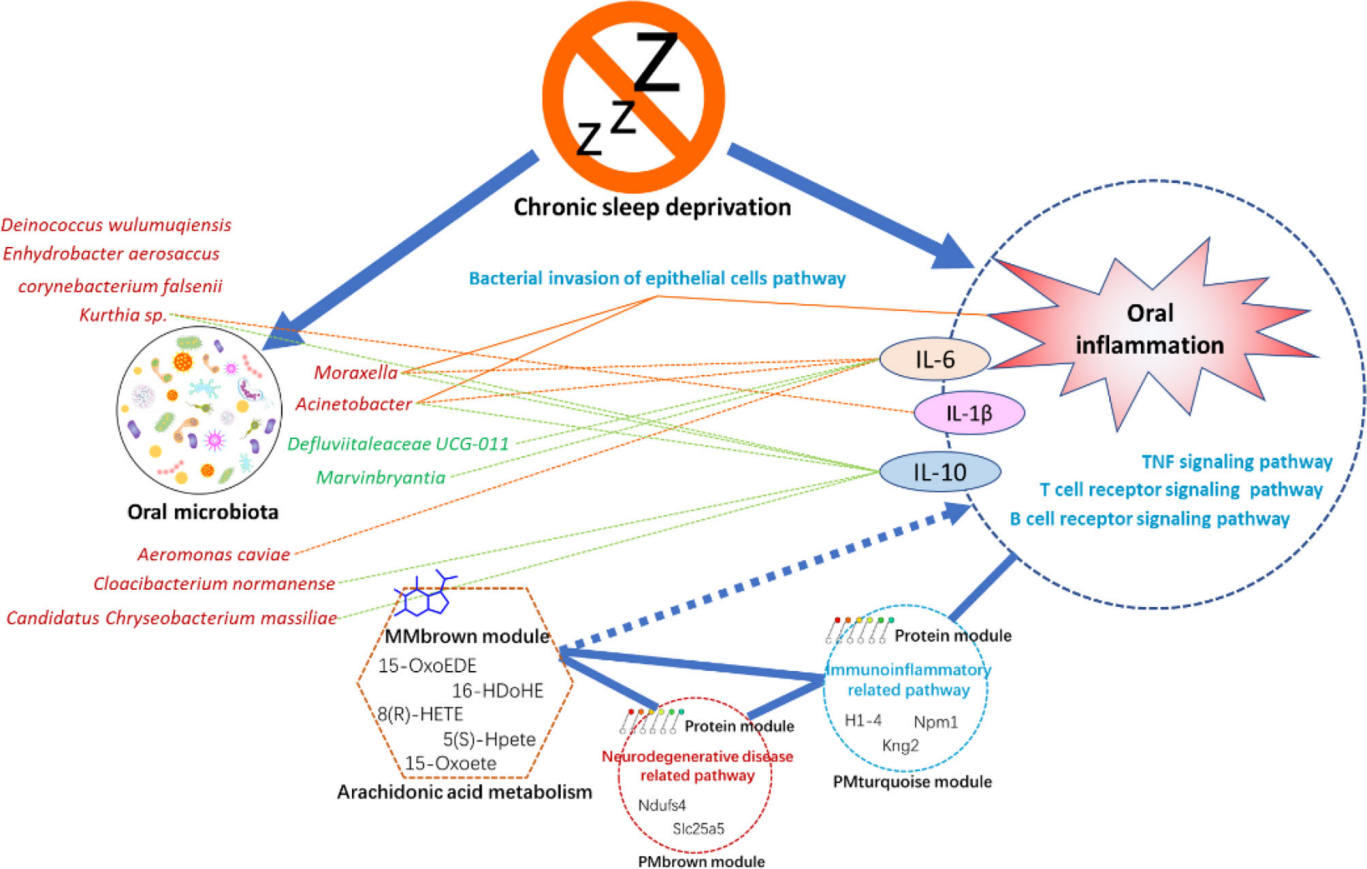
Summaries of the major relationships between CSD and oral homeostasis.

The present study integrated multi-omics analysis of oral microbiome, serum metabolome, and tongue proteome in rats with chronic sleep deprivation (CSD). Multi-omics integration analysis enhanced our ability to focus on the systemic relationships between oral homeostasis and CSD in rats. **Figure 9** illustrates a summary diagram showing the major relationships between CSD and oral homeostasis. In this study, we focused on the effects of CSD on oral inflammation. Histopathology results indicated that CSD led to an inflammatory histological appearance, and immunohistochemistry analysis indicated that the expression of IL-6 and IL-1β in epithelial tissue was elevated, which suggested that CSD could lead to oral inflammation.

We found that CSD increased the serum levels of pro-inflammatory cytokines including IL-1β and IL-6, which also are consistent with the findings described in previous studies (33, 34). The anti-inflammatory cytokine, IL-10, was decreased in serum, suggesting sleep deprivation-induced inflammation also be mediated through a decrease of anti-inflammatory cytokine levels. Serum metabolic analysis demonstrated major perturbations occurred in arachidonic acid metabolism and glycerophospholipid metabolism. Arachidonic acid metabolism was reportedly involved in the pathogenesis of kidney inflammation (35). The metabolites in arachidonic acid metabolism are various polyunsaturated fatty acids, and also termed eicosanoids which are linked to immunity function (36) and triggered different inflammatory responses (35, 37). In this study, docosahexaenoic acid has been reported to exert anti-inflammatory and antioxidative activity (38), which was decreased in CSD rats. However, almost no studies on the association between polyunsaturated fatty acids and oral inflammation could be found. Certain polyunsaturated fatty acids have shown immunomodulatory and anti-inflammatory activity in a variety of inflammatory human diseases including those affecting the intestinal mucosa which together with the oral mucosa belong to the digestive system (15). Glycerophospholipid metabolism reportedly played an important role in many chronic inflammatory diseases including psoriasis (39), Behçet’s disease (40), and arteriosclerosis (41). It has been reported that glycerophospholipids might be significantly related to the anti‐inflammatory activities of diarylheptanoids (42). Taken together, our data indicated CSD led to chronic inflammation in rats.

Previous studies have suggested that sleep deprivation affects hormonal secretion, including that of the HPA axis (43). Our data indicated that CSD also activated the HPA axis evidenced by excessive production of ACTH and corticosterone. According to the metabolic analysis, the steroid hormone biosynthesis pathway was also found to be involved in the perturbation resulting from CSD, and corticosteroid metabolites including corticosterone, 21-deoxycortisol, and 19-hydroxydeoxycorticosterone were upregulated in CSD rats. The activation of HPA axis could lead to the release of cortisol, which promoted an inflammatory immune state and increases the oral inflammatory load (44). Meanwhile, CSD resulted in excessive production of triiodothyronine (T3), showing the HPT axis was activated by CSD, which was consistent with a previous study (45). A previous study indicated that both oral ulcerative mucositis and elevated T3 are related to posttraumatic stress disorder (46). Oral inflammation and the activation of the HPA axis co-occurred frequently in certain diseases (47–49). Whether the imbalances of the HPA and the HPT axis contribute to of exacerbation oral inflammation deserves further study.

According to our data, chronic sleep-deprived rats exhibited decreased serum levels of GABA and 5-HT. Previous studies showed that neurotransmitters in different regions of the brain in mice subjected to sleep deprivation have different variations (50). However, the levels of GABA and 5-HT in whole-brain tissue showed no change in the present study showing that the total levels of GABA and 5-HT in the whole brain have no change.

In this work, we performed an untargeted proteomics analysis on the tongue tissues. Intriguingly, we found that the response of protein expression in tongue tissues was significantly associated with neurodegenerative diseases including Prion disease, Amyotrophic lateral sclerosis, Huntington disease, Alzheimer’s disease, and Parkinson disease. Another interesting focus is immune inflammatory responses occurred in the tongue tissues. We found that Proteasome, T cell receptor signaling pathway, B cell receptor signaling pathway, and TNF signaling pathway were involved in the oral inflammation resulting from CSD. The changes of proteomic profiling expression caused by CSD in tongue tissues were enriched in both neurodegenerative diseases pathways and immune/inflammation-related pathways. CSD induced an inflammatory response and a perturbation of the neurodegenerative disease pathways in tough tissues. Previous studies have suggested that there are associations between oral health and Alzheimer’s disease (47, 51, 52). However, the mechanisms of crosslink between oral inflammation and neurodegenerative diseases are poorly clarification.

It has been reported that the immune response to microbial recognition and interaction on mucosal surfaces may involve in the molecular mechanism underlying the persistent state of age-related inflammation (15). Periodontal pathogens can invade directly or stimulate the immuno-inflammatory response that extends to the systemic circulation, which plays a critical role in the development or exacerbation of certain diseases (53). In the case of dysbiosis, the balance of the oral microbiome ecosystem is disrupted, allowing for an increase in disease-promoting bacteria, leading to conditions such as gingivitis, periodontitis, and caries (54). Therefore, disturbances to the composition of the oral microbiome through external factors can have a significant impact on human health. To assess the effects of CSD on oral microbiota, the third-generation sequencing (TGS) technology was used to evaluate the changes of the microbial abundances in the oral cavity of rats. We found that the *α* diversity of the oral microbiome was not altered, but instead changed with the abundance of specific bacterial taxa in CSD rats. In this study, we noted that the bacterial invasion of the epithelial cells pathway was significantly enriched, suggesting that the changes of oral microbes likely affected the protein expression in the tongue. The abundances of g_*Acinetobacter*, *Candidatus Chryseobacterium massiliae*, and g_*Moraxella* were elevated in CSD rats. Spearman correlation analysis indicated that these three microbes were significantly correlated with multiple proteins in the bacterial invasion of epithelial cells pathway, showing these microbes may trigger the immune responses in the oral mucosa. *Acinetobacter* species are gram-negative organisms and the identification of specific species in the laboratory is a challenge. *Acinetobacter baumannii* is a nosocomial pathogen, which adheres to and invades epithelial cells through the involvement of *Acinetobacter baumannii* outer membrane protein A (55). *Acinetobacter* spp. was reportedly enriched in chronic periodontitis patients (56). *Moraxella catarrhalis* is a human mucosal pathogen related to acute otitis media and chronic obstructive pulmonary disease through the invasion of human epithelial cells (57). Thus, the increased abundance of these three microbial pathogens may be partially responsible for CSD-induced oral inflammation. In addition, oral bacteria have been proposed to play a role in some systemic diseases, including rheumatoid arthritis, cardiovascular disease, adverse pregnancy outcomes, stroke, respiratory tract infection, inflammatory bowel disease, meningitis or brain abscesses, appendicitis, diabetes, and pneumonia (54). Therefore, the perturbation of the oral microbiota caused by CSD may in part contribute to an increased risk of chronic systemic disease.

Multi-omics data integration analysis indicated that PMturquoise and MMbrown, two inflammation-related modules, are strongly correlated. Among the protein modules, PMturquoise and PMbrown have the strongest correlation, which respectively associated with immune-inflammatory responses and Alzheimer’s disease, suggesting there is a strong association between oral inflammation and neurodegenerative diseases. Changes in protein expression in tongue tissue caused by CSD are closely associated with both inflammation and neurodegenerative diseases, providing new insight into the relationship between oral inflammation and neurodegenerative diseases. Some factors, such as CSD, may lead to both oral inflammation and neurodegeneration, with no clear sequential cause-and-effect relationship between the two. Our data strengthen the evidence for a possible link between oral inflammation and neurodegenerative diseases. We obtained the key nodes of each module network by ranking the network node. Histone H1.4 (H1-4), a top-ranked protein in the PMturquoise network, was reported to activate immune defenses and drive further degeneration during central nervous system degeneration and killed neurons through mitochondrial damage and apoptosis (58). Gtsm2, a top-ranked protein in PMbrown network, could reduce potentially deleterious peroxides in cellular anti-oxidant defense processes and ameliorate inflammation (59). As a crucial proinflammatory factor, Bgn (a top-ranked protein in PMblue network) can promote inflammation through TLR2 and TLR4 signaling, thereby enhancing the production of TNF-α and MIP-2 (60). These key nodes in the network may play a more important role in the oral homeostasis disruption caused by CSD.

There has been enormous interest in the possibility that periodontal disease and other oral inflammatory diseases influence this systemic pathological process. Oral inflammation was associated with elevated systemic inflammation and has been linked with increased risk of diabetes (49), Alzheimer’s disease (61), Parkinson disease (62). The inflammation hypothesis suggests that local oral inflammatory conditions are associated with dysbiosis in local ecosystems which are, in turn, related to inflammation beyond the oral cavity. Analysis of the oral cavity and its microbiome may be a useful tool to evaluate chronic systemic diseases that have oral inflammation, such as rheumatoid arthritis and Alzheimer’s disease. Therefore, the risk to human health caused by CSD may be related to oral dysbiosis. Targeting oral dysbiosis and oral inflammation to treat certain systemic diseases may be an effective complementary treatment option in the future.

## Conclusion

Our study systematically investigated the effects of CSD on oral homeostasis and discussed their possible impact on systemic diseases. Integrating proteomics, metabolomics and microbiomics techniques uncovered the systemic relationships between oral homeostasis and CSD in rats. We found that CSD could lead to oral inflammation and subtle changes on oral microbiota composition. Metabolomics and biochemical assay results indicated that CSD led to chronic inflammation and the activation of HPA axis. Meanwhile, the changes of proteomic profiling expression caused by CSD in the tongue tissues were enriched in neurodegenerative diseases pathways and immune/inflammation-related pathways. Multi-omics analysis indicated that the inflammatory response-related modules were significantly correlated with the neurodegenerative disease-related module. Our study is helpful to further understand the role that oral homeostasis plays in the process by which CSD affects human health and disease.

## Data Availability Statement

The datasets presented in this study can be found in online repositories. The names of the repository/repositories and accession number(s) can be found in the article/**Supplementary Material**.

## Ethics Statement

The animal study was reviewed and approved by Institutional Animal Care and Use Committee, Sun Yat-sen University.

## Author Contributions

PL, HY, and WS provided the concept and designed the experiment; PC, JZ, HW, WF, and ZC performed the experiments; PC, HW, and YW analyzed the data; PC and PL wrote the manuscript. All authors have read and approved the final manuscript.

## Funding

Financial support was provided by the Science and Technology Planning Project of Guangzhou, China (No. 201803010082) and Guangdong Academic of Sciences Special Project of Science and Technology Development (No. 2016GDASRC-0104).

## Conflict of Interest

The authors declare that the research was conducted in the absence of any commercial or financial relationships that could be construed as a potential conflict of interest.

## Publisher’s Note

All claims expressed in this article are solely those of the authors and do not necessarily represent those of their affiliated organizations, or those of the publisher, the editors and the reviewers. Any product that may be evaluated in this article, or claim that may be made by its manufacturer, is not guaranteed or endorsed by the publisher.
