# Supplementary figures and images for: Multi-Omics Analysis Reveals the Systematic Relationship Between Oral Homeostasis and Chronic Sleep Deprivation in Rats
Source: Front Immunol. 2022 Mar 31;13:847132. doi: 10.3389/fimmu.2022.847132 (PMC9009293; doi:10.3389/fimmu.2022.847132)

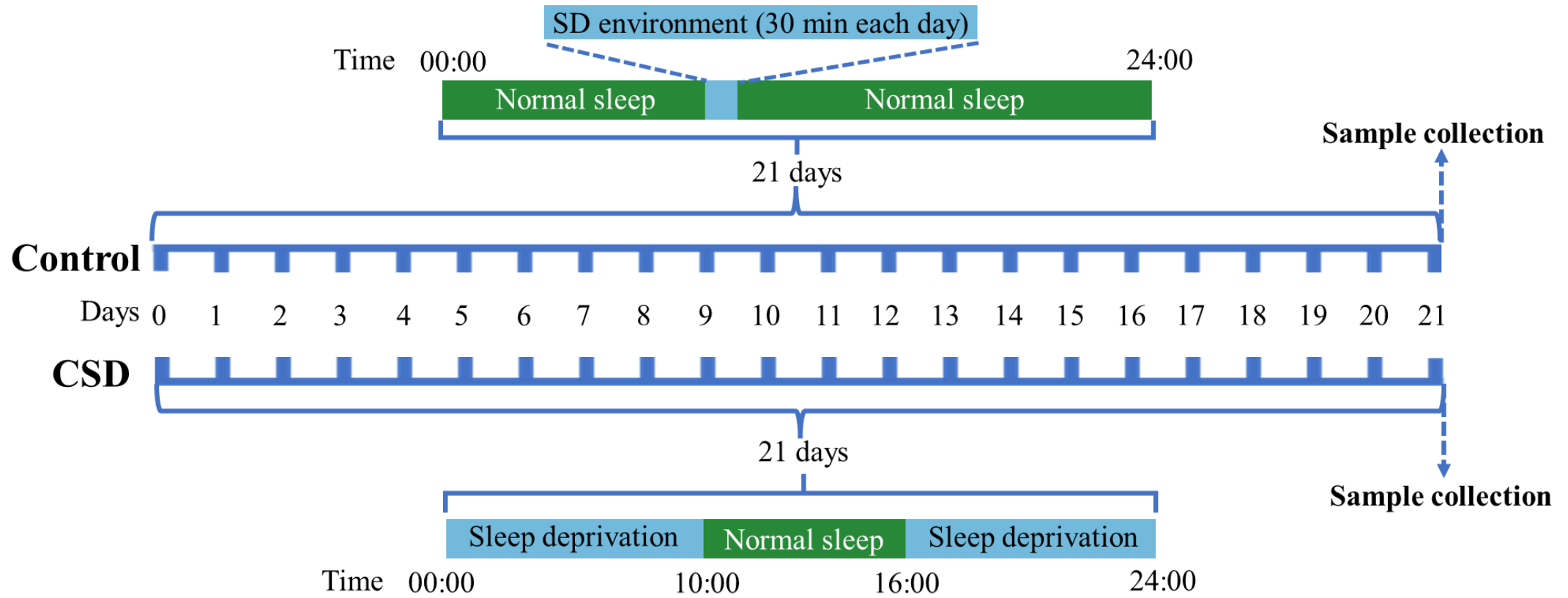

Figure S1. The diagram of the CSD design

Supplement: Supplementary file 1 [file Image_1.pdf]

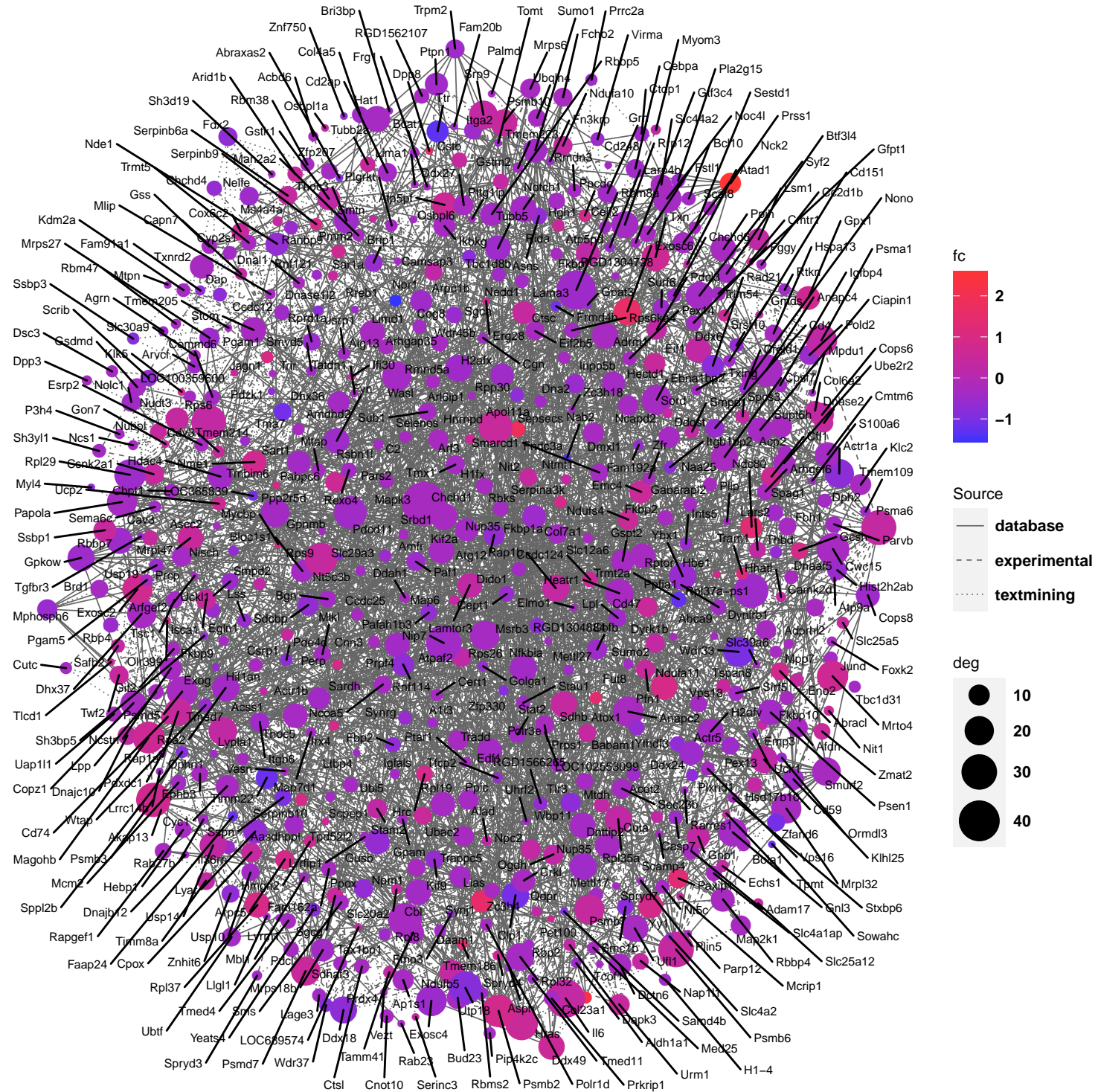

Supplement: Supplementary file 2 [file Image_2.pdf]
